# Silent but Serious: Astrocytoma in a Seven-Month-Old Infant With Atypical Presentation

**DOI:** 10.7759/cureus.114518

**Published:** 2026-08-14

**Authors:** Priscilla Chong, Kulsoom Bilal, Imran Ahmed, Haji Sheeraz Khan

**Affiliations:** 1 Paediatric Department, Hull University Teaching Hospitals NHS Trust, Kingston Upon Hull, GBR

**Keywords:** brain tumour, failure-to-thrive, paediatrics neuro-oncology, persistent vomiting, pilocytic astrocytoma (pa)

## Abstract

Pilocytic astrocytoma (PA) is a common paediatric brain tumour that usually presents with neurological or visual symptoms linked to its location. This report describes a seven-month-old boy with an unusual presentation marked by poor feeding, progressive weight loss, and recurrent vomiting over three months, without early neurological signs. Extensive initial assessments, including feeding evaluations, multidisciplinary reviews, and pharmacological trials, did not reveal a cause. Ultimately, neuroimaging identified a large suprasellar mass with leptomeningeal deposits. Following lumbar puncture, histopathological examination of the biopsy specimen, and molecular testing, the diagnosis of PA was confirmed. Treatment was subsequently initiated with carboplatin and vincristine. Unlike typical PA cases, which often involve headaches, visual problems, or motor deficits, this case underscores that systemic, nonspecific symptoms can signal central nervous system disease. The report highlights the importance of considering neuroimaging in infants with persistent vomiting and failure to thrive to enable earlier diagnosis and potentially improve outcomes in atypical PA presentations.

## Introduction

Pilocytic astrocytoma (PA) is one of the most common central nervous system (CNS) tumours, primarily affecting the paediatric and young adult population, particularly in the first two decades of life [1].

In the United Kingdom, astrocytoma was found to constitute 41% of CNS tumours, with pilocytic astrocytoma comprising 50% of all astrocytomas and 21% of all CNS tumours [1]. It has a favourable prognosis with 5-year survival rates exceeding 90% in both the children and young adult populations [2].

Most common sites affected by this tumour include the cerebellum, optic pathways, hypothalamus, and brainstem. Signs and symptoms are usually dependent on the location of the tumours, but usually include headache, visual deficits, nausea and vomiting, gait imbalances and seizures [1].

This case report focuses on an infant with recurrent admissions in his first year of life due to poor weight gain and failure to thrive. He later developed persistent vomiting, leading to neuroimaging revealing a diagnosis of suprasellar pilocytic astrocytoma. The case highlights an atypical presentation of a common paediatric brain tumour and underscores the diagnostic challenges in infants presenting with non-specific symptoms.

## Case presentation

We present a case of a seven-month-old male infant who had recurrent hospital admissions due to poor weight gain, vomiting, and refusal to feed. He was born at 40+3 weeks of gestation via emergency caesarean section due to abnormalities on cardiotocography. He was the second child of non-consanguineous parents, and there was no significant family history. His birth weight was 4040 g (91st centile), with a head circumference of 35.2 cm (50th centile). His newborn examination was unremarkable.

He was first referred to the paediatric team at 3.5 months of age by his general practitioner after a health visitor noted that his weight had fallen from the 91st centile at birth to the 25th centile.

At his initial clinic review at 4.5 months, he weighed 5.86 kg (9th centile). He was receiving mixed feeding (breast and bottle). No other significant signs or symptoms were identified, and his developmental milestones were appropriate for age. Clinical examination revealed a mild tongue-tie but was otherwise unremarkable. Feeding advice, closer health visitor monitoring, and a follow-up appointment in four weeks were arranged.

However, at 5 months, his weight had dropped further to 5.66 kg (0.4-2nd centile), prompting an urgent referral and admission for management of failure to thrive. A comprehensive assessment, including reviews by the dietetics and speech and language therapy teams, did not identify a clear cause. During this admission, he developed increased vomiting, initially attributed to a rapid increase in feed volume. This improved with the introduction of thickened feeds, and he was discharged shortly afterwards weighing 5.808 kg (0.4-2nd centile).

He was readmitted 2 weeks later, as his weight had fallen again to 5.77 kg and vomiting had become more frequent. There was no concern documented about his previous neurological examinations. He was achieving normal developmental milestones. He had good head and neck control, could roll from front to back and back to front, and was able to sit with support while reaching for toys. He had a social smile and giggled when happy. On this admission, he was 5.5 months old. On examination, his observations were within normal limits. He was alert, conscious, and interactive. Apart from occasional pendular nystagmus, a neurological (including cranial nerves and motor) examination was unremarkable, with no other neurological deficit. No fundoscopy was performed.

A nasogastric (NG) tube was inserted for supplementary feeds; however, vomiting persisted. A videofluoroscopy study was normal. A tongue-tie division was performed, and antiemetic trials were attempted but were ineffective. The NG tube was subsequently replaced with a nasojejunal (NJ) tube, and oral feeds were gradually reintroduced.

**Figure 1:**
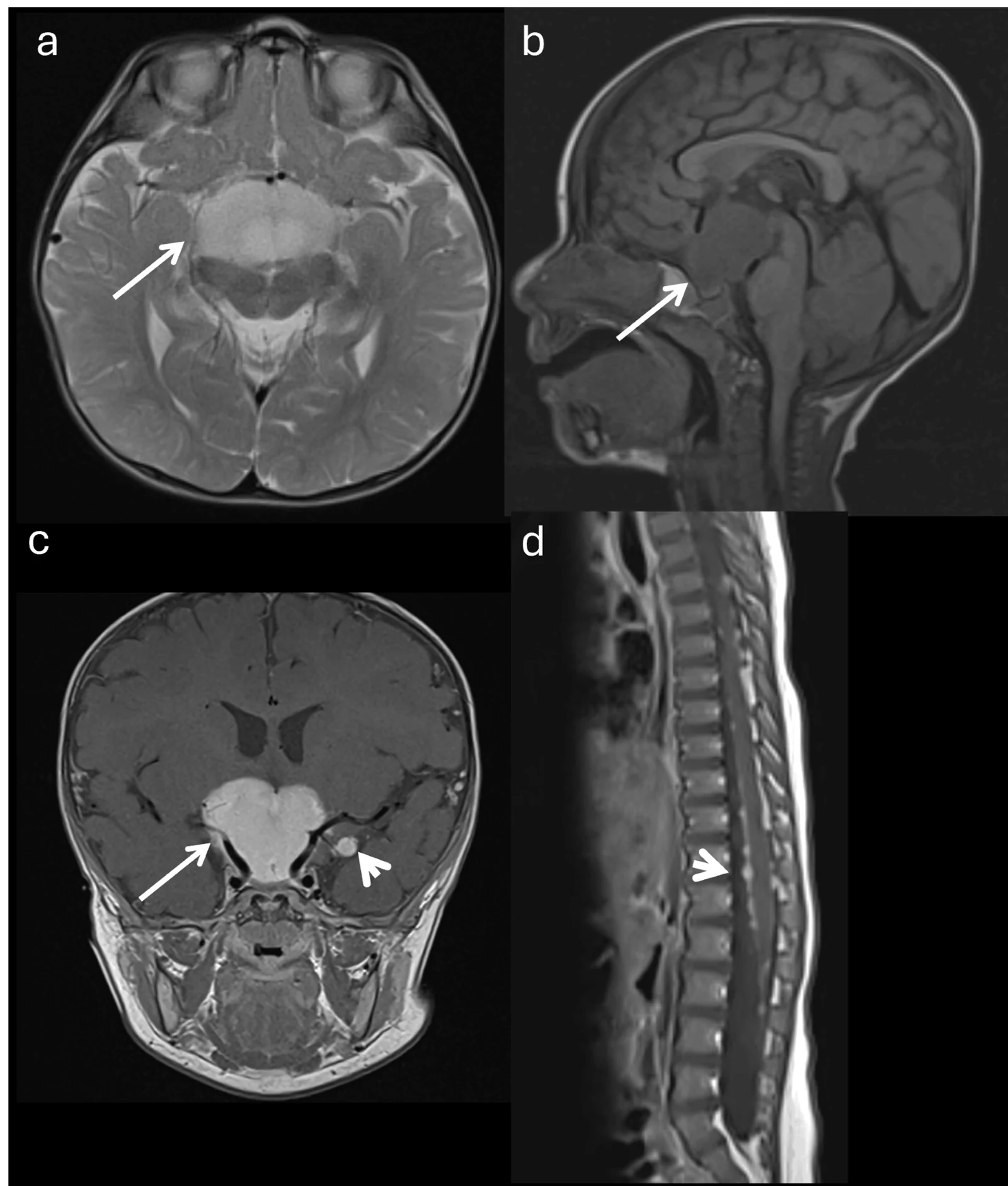
MRI brain and spine showing a suprasellar mass with disseminated disease (a, b) Axial T2 and sagittal T1 images demonstrate a large lobulated suprasellar lesion with mass effect on the third ventricle and brainstem. (c) Coronal post-contrast T1 image shows homogeneous enhancement of the lesion with additional intracranial subarachnoid deposits. (d) Sagittal post-contrast T1 spine image shows nodular leptomeningeal deposits along the cord. Arrows indicate the suprasellar mass; arrowheads indicate deposits. These findings are consistent with a suprasellar pilocytic astrocytoma, which was subsequently confirmed through histopathological assessment and molecular testing.

As vomiting continued despite these interventions, an MRI of the head and spine was arranged to rule out central causes. MRI brain and spine (Figure 1) showing a suprasellar mass with disseminated disease. Axial T2 and sagittal T1 images demonstrate a large lobulated suprasellar lesion with mass effect on the third ventricle and brainstem (Figures 1a-1b). Coronal post-contrast T1 image shows homogeneous enhancement of the lesion with additional intracranial subarachnoid deposits (Figure 1c). Sagittal post-contrast T1 spine image shows nodular leptomeningeal deposits along the cord (Figure 1d). Differential diagnoses included a germ cell tumour, embryonal tumour, or metastatic disease.

Neurosurgical review was obtained, and further imaging and ophthalmologic assessment were arranged. The initial ophthalmology examination was normal; however, he developed horizontal nystagmus several days later. Lumbar puncture revealed cerebrospinal fluid containing "clusters of suspicious atypical cells" on cytology.

The case was referred to a tertiary paediatric oncology centre for multidisciplinary team (MDT) management, where histopathological examination and molecular analysis identified a KIAA1549::BRAF fusion, supporting the neuropathological diagnosis of pilocytic astrocytoma. Treatment with carboplatin and vincristine was subsequently commenced.

## Discussion

PA is the most common low-grade glioma amongst the paediatric population and holds an excellent prognosis following surgical resection [1]. PA lesions are most commonly located in the cerebellum, optic pathway, and hypothalamic/chiasmatic regions, and their presentation can vary greatly according to tumour location, growth pattern and patient age [3]. While PA accounts for 21% of paediatric CNS tumours, presentation with isolated failure to thrive in the absence of neurological signs is exceptionally rare, with fewer than 20 cases reported in the literature to date. Due to this, diagnosis may often be delayed, especially in infants because of their presentation with non-specific symptoms.

The classic symptoms of PA include raised intracranial pressure, focal neurological deficits, seizures, or visual disturbance. However, there is an increasing number of published case reports demonstrating that PA can often present with more subtle symptoms resulting in atypical presentations. Presentations have varied significantly, including seizures, precocious puberty, hydrocephalus, developmental concerns and constitutional symptoms such as vomiting and feeding difficulties. Bell et al. similarly reported an infant presenting with vomiting, feeding difficulty, and weight loss before the diagnosis of PA, supporting the observation that early symptoms may be non-specific [4]. Similarly, Rodrigues et al. [5] and Tosur et al. [6] reported infants in whom failure to thrive and weight loss were among the earliest manifestations of hypothalamic PA, emphasising that constitutional symptoms may precede overt neurological signs. Taken together, these reports suggest that failure to thrive represents an important, albeit uncommon, early manifestation of PA that should prompt consideration of neuroimaging when routine investigations are unrevealing.

Failure to thrive is usually associated with hypothalamic tumours resulting in diencephalic syndrome, characterised by severe weight loss despite a normal/increasing appetite, hyperactivity, and preserved height growth [7]. It is important to note that not all patients with hypothalamic or suprasellar PA will develop all symptoms of diencephalic syndrome. In our patient, the mass effect on the brainstem and third ventricle likely contributed to vomiting through direct compression of the area postrema and vagal nuclei, while hypothalamic involvement may have disrupted satiety signalling, leading to poor feeding. The absence of classic diencephalic features (hyperactivity, preserved linear growth) suggests that only specific hypothalamic circuits were affected, highlighting the heterogeneity of presentation in suprasellar PA. As our patient did not demonstrate the complete syndrome, presenting with persistent failure to thrive and minimal neurological signs in the earlier stages, making diagnosis more challenging. The three-month interval from initial weight faltering to neuroimaging in our patient is consistent with the diagnostic delays reported by Rodrigues et al. (2024) and Tosur et al. (2017) [5,6], suggesting that atypical presentations commonly extend the time to diagnosis. This suggests that clinicians should not rely on the classical constellation of features before considering neuroimaging, as hypothalamic PA may initially present with only isolated weight faltering.

There is a wide variability in atypical PA presentations. Notably, failure to thrive or poor weight gain was the presenting feature in four cases (ours and those of Rodrigues et al., Tosur et al., and Bell et al.), all of whom were under 2 years of age [4-6]. This suggests that constitutional symptoms are a particular diagnostic challenge in infancy. In contrast, older children more commonly presented with headaches, visual disturbances, or focal neurological deficits, even when tumours were in similar locations. This review underscores that age is a critical determinant of presenting symptoms, with infants more likely to manifest systemic rather than localising neurological signs.

Clinicians evaluating infants with persistent failure to thrive should continue to investigate common causes of failure to thrive, including nutritional, endocrine, and gastrointestinal causes. However, if faltering weight persists despite usual treatment, intracranial pathology should be considered. This becomes even more pertinent when accompanied by symptoms of recurrent vomiting, developmental concerns and subtle neurological findings. Earlier recognition of these atypical presentations may reduce diagnostic delay and facilitate timely neurosurgical referral, ultimately improving clinical outcomes.

Although PA has an excellent overall prognosis, the three-month diagnostic delay in our patient raises the question of whether earlier intervention could have prevented the development of leptomeningeal dissemination, which was present at diagnosis. Future studies should examine whether atypical, non-neurological presentations are associated with higher rates of disseminated disease due to delayed recognition. Our case adds to the limited literature on PA presenting as failure to thrive in infancy and reinforces that PA exists on a spectrum of clinical presentations. However, this is a single case report, and the findings may not be generalizable to all infants with failure to thrive. It is not possible to definitively establish causation between the tumour and the feeding difficulties, as the patient had multiple concurrent interventions (e.g., tongue-tie division, feeding modifications) that may have contributed to his clinical course.

Table 1 summarises the case reports describing paediatric PA that were included in this review.

**Table 1:** Summary of published case reports describing paediatric pilocytic astrocytoma presenting with atypical or non-neurological symptoms that were included in this review This table outlines key clinical features of reported cases, including the authors, year of publication, patient demographics, initial presenting symptoms, and anatomical location of the tumour. The cases demonstrate the wide variability in presenting features and tumour sites among paediatric patients with pilocytic astrocytoma.

Author & Year	Age/Sex	Presenting symptoms	Location of Tumour
Bell E, Kanodia AK, Gunaratne B, Edgar A. (2018) [4]	8-month-old male	Two-month history of vomiting, feeding difficulty, weight loss, followed by sleepiness, irritability, tense fontanelle, and upgaze palsy due to communicating hydrocephalus	Thoracic intramedullary spinal pilocytic astrocytoma with intracranial and spinal leptomeningeal dissemination
Rodrigues LM, Lyra L, De Almeida CB, Konstantyner T. (2024) [5]	8-month-old	Presented with poor weight gain and later developed hiccups, myoclonus, and vomiting	Brainstem
Tosur M, Tomsa A, Paul DL. (2017) [6]	2-year-old female, 10-month-old male	Both presented with poor weight gain; the second patient later presented with diarrhoea and vomiting followed by nystagmus	Hypothalamus, suprasellar
Nugraha HG, Yoasta E, Sobana M, Usman HA. (2025) [8]	7-year-old, female	Presented with a 1-month history of loss of vision and had 6 months of continuous headaches, 3 months of postural imbalance, and 4 months of blurred vision	Right cerebellum
Alkhaibary A, AbuHassan A, Azzubi M. (2025) [9]	8-year-old, female	Presented with 1 week of left-sided upper and lower limb weakness, facial deviation, headaches, and vomiting	Right midbrain
Pramantara I, Satyarsa A, Golden N, Wayan N, Mahadewa T, Maliawan S, Suarjaya I. (2024) [10]	7-year-old, male	Presented with left eye proptosis and loss of vision over 2 years	Left pars intraorbital to pars intracranial
Al-Mousa SS. (2024) [11]	3-year-old, female	Presented with 2 weeks of persistent headaches, vomiting, and difficulty walking	Right cerebellopontine angle
Mahakul DJ, Premsagar IC. (2023) [12]	11-year-old, male	Presented with headache and hearing impairment in the right ear	Right cerebellopontine angle
Gala F, Agrawal H, Chawla P, Andar U. (2023) [13]	5-year-old, male	Presented with 1.5 months of difficulty in walking and standing, headache, and vomiting	Right pons, vermis, and both cerebellar hemispheres
Offenbacher R, Kobets A, Dalvi N, et al. (2023) [14]	9-month-old, male	Presented with 4 months of regression of milestones and constipation	Spinal
Ferriastuti DW, Fauziah D, Fatmariyanti S. (2022) [15]	10-year-old, male	Presented with left eye tumour for 3 years, with 1 year of intermittent redness and watering of the left eye and reduced vision	Left intraconal
Vegi O, Mouza A, Akmal T, Fadhlan R, Husain S. (2022) [16]	8-year-old, female	Presented with loss of consciousness 2 days prior to admission; had a 1-week history of headaches	Left temporal-parietal
Zakaria Z, Raja Mohd Rasi RZ, Rahman NAA. (2022)[17]	14-year-old, male	Presented with hypertension, headache, and blurred vision	Right cerebellopontine angle
Mirone, G, Schiabello, L, Chibbaro S, Bouazza S, George B. (2009) [18]	12-year-old, male	Presented with progressive hearing loss and headache	Right cerebellopontine angle arising from the VIII nerve complex
Çatlı G, Abacı A, Anık A, et al. (2014) [19]	2 years 9 months, male	One-month history of headache, vomiting, and gait disturbance; Later developed central precocious puberty before rapid enlargement of the suprasellar lesion	Suprasellar juvenile pilocytic astrocytoma (initially diagnosed as hypothalamic hamartoma)
Singuepiré A, Coulibaly OS, Beydari BH, et al. (2024) [20]	9-year-old male	Generalised tonic-clonic seizures from 6 years of age with increasing seizure frequency; occasional headache.	Right parietal (supratentorial) pilocytic astrocytoma with cystic and calcified components

## Conclusions

Pilocytic astrocytoma is a common paediatric brain tumour, with clinical presentation largely determined by tumour location and size. This case of a seven-month-old presenting primarily with poor weight gain and feeding difficulties highlights the importance of maintaining a high index of suspicion for intracranial pathology in infants with failure to thrive, even in the absence of classic neurological or ophthalmological signs. It contributes to the growing literature on atypical presentations of infantile pilocytic astrocytoma, where systemic symptoms precede overt neurological manifestations. Early consideration of neuroimaging in such cases may enable prompt diagnosis, guide timely intervention, and potentially improve clinical outcomes.
